# Pregnancy log records and management of risk factors for gestational diabetes mellitus reduces its incidence

**DOI:** 10.3389/fpubh.2025.1645835

**Published:** 2025-09-03

**Authors:** Tianzi Li, Qi Zhang, Lili Ma, Xiaoxiao Peng, Yan Li, Fengli Song, Xiuhua Ma

**Affiliations:** ^1^Capital Medical University Daxing Teaching Hospital, Beijing, China; ^2^School of General Practice and Continuing Education, Capital Medical University, Beijing, China

**Keywords:** gestational diabetes mellitus, pregnancy, log records, risk factors, lifestyle, compliance

## Abstract

**Objective:**

To explore the preventive effect of pregnancy log records and management and the influence of maternal lifestyles on the incidence of gestational diabetes mellitus (GDM).

**Design:**

This case-control study was conducted at a hospital in Beijing, China.

**Population or sample:**

A total of 348 pregnant women who underwent normal early screening for GDM were included in this study between April and November 2022.

**Methods:**

All enrolled pregnant women were educated for GDM and were assigned to an observational group and a control group via a random number-generating table. The two group received routine guidance during pregnancy. The observational group kept individualized log records and management independently from 14 to 28 weeks of gestation.

**Main outcome measures:**

Both groups were subjected to a 75-g oral glucose tolerance test at 24–28 weeks of gestation, and their incidence rates of GDM were compared.

**Results:**

The incidence rates for GDM in the second trimester was 9.5% (15/158) in the observational group and 20.5% (34/166) in the control group, with the differences statistically significant (*p* < 0.05). In terms of lifestyle, exposure to passive smoking, weight gain, the frequency of eating out and physical activity per week, and average sleep duration were independent risk factors for GDM (*p* < 0.05).

**Conclusion:**

Log records and management have an effective impact on reducing the incidence of GDM. Dietary habits, physical activity, sleep patterns, exposure to passive smoking, and weight gain during pregnancy were among the risk factors associated with GDM. Maternal lifestyle was a critical determinant for the occurrence.

## Introduction

Gestational diabetes mellitus (GDM) is a common obstetric complication of pregnancy onset and principally refers to normal glucose metabolism before pregnancy, and the emergence or diagnosis during pregnancy of a type of high-risk pregnancy (1). In 2017, the main risk factors for GDM cited by the American College of Obstetricians and Gynecologists (ACOG) always included a variety of aspects (2).

Blood glucose levels in pregnant women are closely correlated with diet and exercise. An increasing number of studies have shown that reducing or improving the risk factors for GDM is useful, which includes health consultation, dietary adjustments, intensive exercise, nutrient supplementation, and pharmaceutical interventions (3)etc. Due to significant differences in the timing of intervention, interventional methods, and observational indicators, the results of studies often vary or even contradict one another. Research has shown that reasonable diet or exercise interventions harbor a positive preventive effect on GDM (4). In contrast, Rogozinska’s study revealed that dietary intervention exerted a positive effect on reducing GDM, but that combined dietary and exercise interventions did not reduce the risk of GDM (5). However, Bain (6) and Ruifrok et al. (7) analyzed a group at high risk for GDM applying the Theory of Planned Behavior (TPB) as a framework in order to construct interventions and implement health education to improve women’s awareness of GDM.

It is imperative to gain a comprehensive understanding of disease risk factors in early pregnancy, and to promote active adherence to risk-factor management (8). We now acknowledge the hazards associated with risk factors, treatment, and improvement based on the comprehensive measurement on type-2 diabetes. This comprehensive approach involves dietary and exercise interventions, the use of medications, and monitoring of blood sugar level etc. The early control of risk factors for GDM may reduce its incidence, although previous studies in this area have proven to be inadequate. To control the onset of GDM, risk factors for GDM such as hypertension and polycystic ovary syndrome (PCOS) should be targeted with effective interventions. The maternal lifestyles should be changed accordingly (9).

We expect to explore the preventive effects of personal log records and management on the onset of GDM in the middle trimester in pregnant women with a high risk of GDM, and to better understand the influence of maternal lifestyles on the incidence of GDM.

## Methods

### Participants

We enrolled a total of 1,215 pregnant women with complete perinatal health records and who received regular hospital care. According to the inclusion and exclusion criteria, the research participants were determined. Informed consent was obtained from a total of 348 pregnant women with normal early-pregnancy diabetes screening between April 2022 and November 2022 at the Capital Medical University Daxing Teaching Hospital in Beijing, China.

**Figure 1:**
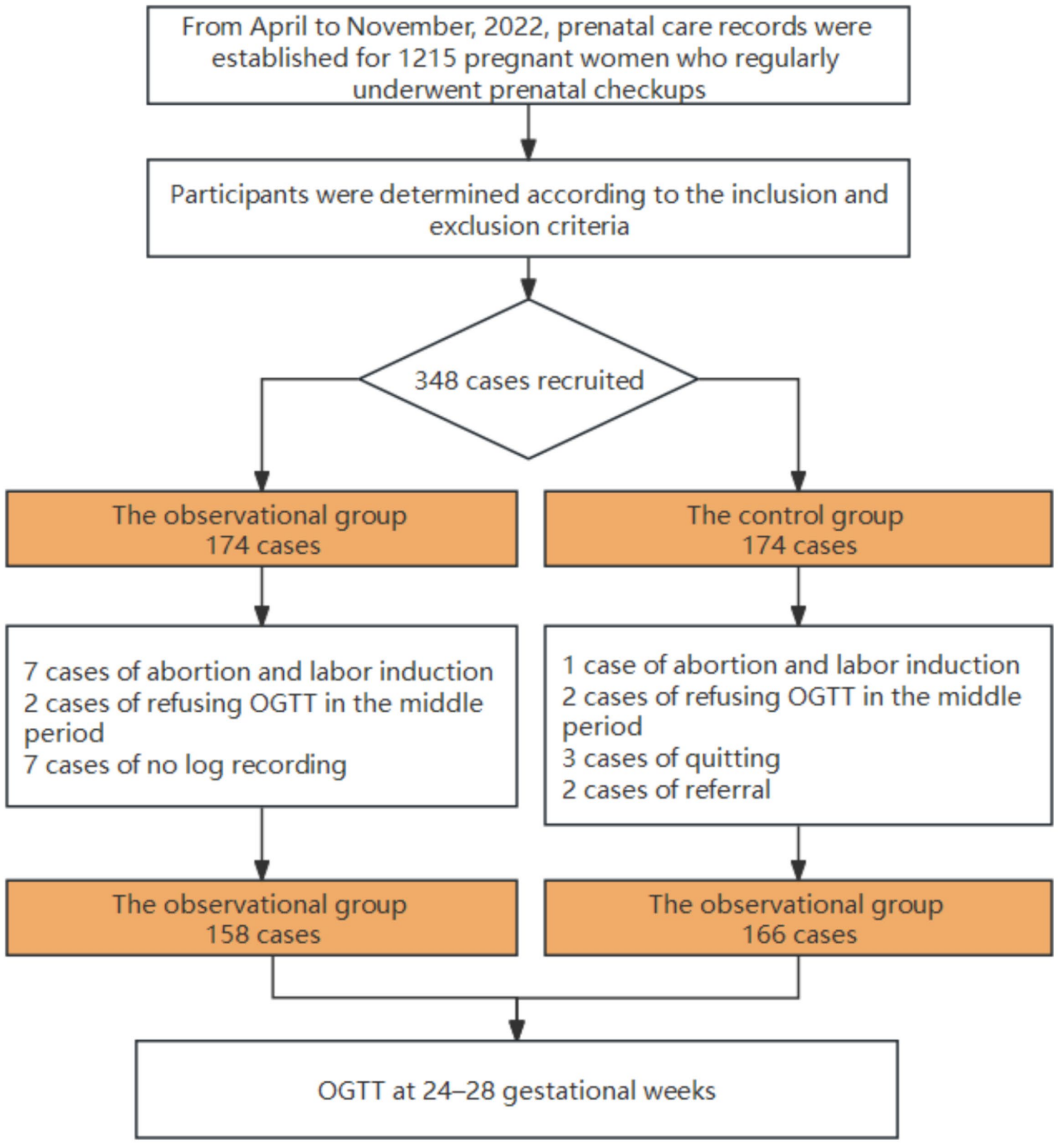
Screening, inclusion, and randomization of subjects.

After their study instructions, the participants were assigned using a random- number generating table and observed. There were initially 174 patients in the experimental group and 174 patients in the control group (1,1). Due to miscarriages, induced labor, hospital transfer, or delivery at a foreign location, some women withdrew from the groups. A total of 17 patients were excluded from the control group for reasons such as glucose tolerance screening during the second trimester, and 7 patients in the experimental group refused to undergo log recording according to the standard observational process. Ultimately, data from 158 patients in the observational group and 166 patients in the control group were collected for analysis. Screening, inclusion, and randomization of subjects in this study are illustrated in Figure 1.

Inclusion criteria were as follows:

(1) Pregnant women who established their files and received regular prenatal care in the early stages of pregnancy; (2) singleton pregnancies; (3) women with fetuses whose gestational age was less than 14 weeks at the time of enrollment; (4) women who possessed one or more of the following high-risk factors: age >35 years, body mass index (BMI) ≥ 28 kg/m^2^, a family history of diabetes, family history of hypertension, history of delivering a large baby, history of gestational diabetes, history of gestational hypertension, history of hypertension, positive for urine glucose, hyperlipidemia, hypothyroidism, PCOS, irregular menstrual cycles before pregnancy; (5) women who had been screened for pre-pregnancy diabetes or glucose intolerance via early screening; and (6) women who were willing to cooperate with management.

Exclusion criteria were as follows:

(1) Twin and multiple pregnancies; (2) mentally or emotionally unstable individuals who could not cooperate; (3) individuals who had taken medications that might affect glucose and lipid metabolism during pregnancy; (4) individual carriers of active hepatitis or hepatitis B virus; (5) individuals with a history of chronic diseases such as heart disease, kidney disease, respiratory system disease, immune system disease, or connective tissue disease; and (6) individuals who were noncompliant to write and would not cooperate with improving guidance.

Withdrawal criteria were as follows:

(1) Pregnancy not reaching 24 weeks for various reasons; (2) poor compliance, with a completion rate of the pregnant woman’s diary ≤10%.

The sample size was calculated by formula *n* = (U*_α_* + U*_β_*)^2^(1 + 1/k)P(1-P)/(P1-P2)^2^ (10). According to the literature (11, 12), P1 = 33%, P2 = 20.7%, P = (P1 + P2)/2, α = 0.05, and *β* = 0.20. Upon consulting the table, U_α_ = 1.6449, U_β_ = 0.8416, and n1 = n2 = 158. Considering that 10% would be lost to follow-up, *n* was at least 348.

### Measures

For this study we obtained the ethical approval of the Medical Scientific Research Ethics Committee, Beijing Daxing District People’s Hospital (No.20220412LLKYLX-1-05), and completed clinical trial registration in China (ChiCTR2200059695).

All enrolled pregnant women were instructed as to individualized, controllable risk factors for GDM and then allocated to an observational group and a control group via a random-number generating table from SPSS software. Simple randomization was used to identify the two groups of participants, each research subject can be equally likely to be assigned to either of the two different groups. Individualized health instruction was provided to the study subjects one-on-one at the time of enrollment, and after completion, they were randomly assigned to the two groups as above. Regarding the gestational age, and the experts’ experience, individualized suggestions were formulated, and there were differences among individuals. A total of 174 subjects were assigned to the observational group, and 174 were assigned to the control group. We collected the basic information of all patients, and recorded the number of risk factors for GDM in each patient.

The control group received individualized health instruction and nutritional guidance on the day of enrollment, followed by conventional healthcare on a regular basis. The observational group, in addition, was required to record daily logs related to GDM risk factors and submit these results weekly. Targeted intervention plans were then provided based upon the recorded information. For different pregnant women at different gestational weeks, various intervention measures were proposed.

The observational group was given a corresponding record form according to the pregnant women’s own risk factors for GDM, informed in detail how to fill in the form, and asked to record details each day. Be part of paper diary, specific forms included diet and weight records, exercise-monitoring tables, sleep records, and blood-pressure monitoring tables. Besides, with the assistance of technology and digital software, the information regarding the conditions of pregnant women and their fetuses could be efficiently stored and recorded. At a fixed time each week, pregnant women were reminded to maintain a log through WeChat or telephone follow-up, sustain long-term communication, and solve emrging problems in real time. A pregnant woman was recorded 1 week after the photograph was sent to the assistant for WeChat, and content was adjusted according to the recorded content and fetal growth during the pregnancy week or changes in the condition of the corresponding content. The main evaluation is targeted at the log-recorded status and contents. For instance, based on the gestational weeks and prenatal examination results of different pregnant women, the timing and methods of exercise for these women could be adjusted accordingly.

The two groups received routine guidance during pregnancy. Including regular prenatal check-ups and outpatient health education, which were universally applicable. The observational group conducted individualized recording of diet, physical activity, weight, blood pressure, and related risk factors from 14 to 28 gestational weeks of pregnancy. These women were required to record their pregnancy log independently, and proposed targeted intervention plans according to their record content in the next pregnancy health-care period (The pregnancy implementation schedule is listed in Table 1).

**Table 1:** Pregnancy implementation schedule.

Items	12 ~ 12^+6^W	13 ~ 19^+6^ W	20 ~ 23^+6^W	24 ~ 28 W
Questionaire	√			
Basic information	√			
Screening for GDM risk factors	√			
Prenatal education normal directions	√			
Check pregnant women’s behavioral records		√	√	√
Record lecture time	√			
OGTT	√			√

√ represents action performed.

After at least 3 months of follow-up, the two groups were compared with respect to their 75-g glucose tolerance test (OGTT) results at 24–28 gestational weeks and weight gain at 28 weeks. The incidence of GDM in the two groups was also compared, and the effects of weight on GDM in the second trimester and the effects of compliance on plasma glucose outcomes in the observational group were analyzed.

Among which, compliance means consistency in following medical physicians’ instructions. For example, the frequency of keeping a diary or recording a log, the frequency of taking medicine, etc. In this study, The average recording time of 6–7 days per week was classified as “better” in the observational group from 14 to 28 weeks of gestation, that of 4–5 days was classified as “good,” that of 2–3 days was classified as “moderate,” and that of 1 week and below was classified as “poor.”

### Data analysis

All data from pregnant women were collected by specific individual and data were entered by two individuals; and the completeness and accuracy of the data were evaluated regularly to ensure their validity and accuracy. We adopted SPSS 25.0 software for statistical analysis of the general data derived from our research subjects. The measurement data that conformed to a normal distribution were described by mean ± standard deviation(x ± s), and comparisons between groups were conducted by independent-sample t-test. Measurement data that did not follow a normal distribution were described by M (P25, P75), and comparisons between groups were executed with the Mann–Whitney U test. Counting data were described as a frequency and percentage, and the equilibrium test was performed using the χ^2^ test for inter-group comparisons. Factors were analyzed via logistic regression analysis. The significance level of the statistical test variables was considered to be significant at *p* < 0.05.

## Results

### Clinical data of the two groups

The general information of the observational and control groups was as follows: the mean age of the former group was 30.77 ± 4.05 years, the pre-pregnancy BMI was 22.88 (20.89, 26.24) kg/m^2^, the enrolled gestational age was 12 (12, 13) weeks, the number of pregnancies was 0 (0, 1), and the production time was 0 (0, 1) times. The mean age of the latter group was 31.12 ± 3.97 years, the pre-pregnancy BMI was 22.6 (20.69, 24.91) kg/m^2^, the enrolled gestational age was 12 (12, 13) weeks, the number of pregnancies was 1 (0, 2), and the production time was 0 (0, 1). Age, pre-pregnancy BMI, pregnancy status, educational level, occupation, personal monthly income, and early diabetes screening results were not significantly different between the two groups (*p* > 0.05).

Weight gain in the observational group was 6 (4, 8.5) kg and 7.45 (5, 10) kg in the control group, with the difference in weight gain statistically significant (*p* < 0.05). However, body weights did not differ (*p* > 0.05; see Table 2 for details).

**Table 2:** Clinical data of the two groups.

Items	The observational group (*n* = 158)	The control group (*n* = 166)	Statistical value	*p*-value
Age (years)	30.77 ± 4.05	31. 12 ± 3.97	0.768a	0.443
Height (m)	1.62 ± 0.06	1.62 ± 0.05	−0.681^a^	0.496
Prepregnancy weight (kg)	61.9 (54, 70)	60 (53.25, 66)	−1.572^b^	0.116
Prepregnancy BMI (kg/m^2^)	22.88 (20.89, 26.24)	22.6 (20.69, 24.91)	−1.345^b^	0.179
Gestational age	12 (12, 13)	12 (12, 13.5)	−0.687^b^	0.492
Gravidity	0 (0, 1)	1 (0, 2)	−0.74^b^	0.459
Parity	0 (0, 1)	0 (0, 1)	−1.685^b^	0.092
Early FPG (mmol/L)	4.5 (4.2, 4.7)	4.5 (4.3, 4.8)	0.812^b^	0.417
Early 1 h PG (mmol/L)	7.4 ± 1.5	7.3 ± 1.5	0.753a	0.452
Early 2 h PG (mmol/L)	6.2 (5.6, 7.2)	6.6 (5.8, 7. 1)	1.467^b^	0.142
Weight gain at 28 weeks of pregnancy (kg)	6 (4, 8.5)	7.45 (5, 10)	3.213	0.001

^aRepresents the F value, and bRepresents the Z value of the Mann–Whitney test.^

### Incidence of GDM

The incidence of GDM in the second trimester was 9.5% in the observational group (*n* = 158), significantly lower than the 20.5% in the control group (*n* = 166) (*p* < 0.05).

### Lifestyle effects

We evaluated the effects of dietary habits, physical activity, sleep patterns, environmental effects, and body weight on GDM during pregnancy. The number of individuals eating outside the home per week, the number of individuals physical activity per week during pregnancy, the average sleep duration, the women exposed to active and passive smoking, and those experiencing weight gain differed in comparison to those in the non-GDM group (*p* < 0.05). In the multivariate logistic regression analysis, potential confounding variables such as age, gestational age, gravidity and parity were included to adjust for their effects on the outcome. Passive smoking, weight gain, the number of people eating per week, the number of individuals exercising per week during pregnancy, and average sleep duration were independent risk factors for GDM (*p* < 0.05).

The correlation analysis of multiple influencing factors and the onset of GDM is depicted in Table 3.

**Table 3:** Correlation analysis of influencing factors and the onset of GDM.

Variables	β	S. E.	Wald *χ*^2^	*p*-value	The odds ratios	95%*CI*
Frequency of meals eaten out per week(times)						
0				Reference		
1 ~ 2	−0.79	0.799	0.977	0.323	0.454	0.095 ~ 2.13
≥3	1.104	0.482	5.246	0.022	3.016	1.173 ~ 7.757
Weight gain at 28 weeks(kg)	0.044	0.013	10.837	0.001	1.045	1.018 ~ 1.072
Frequency of physical activity per week during pregnancy(times)						
0	0.959	0.328	8.539	0.014	2.608	1.371 ~ 4.96
1 ~ 2	−0.985	0.46	4.576	0.003	0.105	0.151 ~ 0.921
≥3				Reference		
Average sleep duration						
<7 h				Reference		
7 ~ 9 h	1.504	0.846	3.158	0.076	4.5	0.857 ~ 23.641
>9 h	−1.504	0.782	3.702	<0.001	1.56	0.076 ~ 1.622
Smoking	0.948	1.373	0.477	0.913	0.864	0.063 ~ 11.791
Exposure to passive smoking	0.702	0.418	2.819	0.032	2.019	0.889 ~ 4.583

### Compliance analysis

The evaluation of compliance is also a manifestation of the log record evaluation. With regard to the analysis of compliance in the observational group, of the 158 study subjects, 78 (49.4%) manifested better compliance, 44 (27.8%) exhibited good compliance, 28 (17.7%) showed moderate compliance, and eight (5.1%) displayed poor compliance. Maternal compliance was correlated with the onset of GDM (*p* < 0.05), and Table 4 shows this relationship in the observational group.

**Table 4:** Relationship between compliance and GDM onset in the observational group.

Variable	β	S. E.	Wald *χ*^2^	*p*-value	The odds ratios	95%CI
Compliance						
Better (6–7 days/week)				Reference		
Good (4-5 days/week)	1.299	1.24	1.098	3.667	0.295	0.323 ~ 41.638
Moderate (2-3 days/week)	3.597	1.085	10.993	36.474	0.001	4.351 ~ 305.731
Poor (0–1 days/week)	3.833	1.244	9.501	46.2	0.002	4.038 ~ 528.592

The reasons for poor compliance in the observational group included severe morning sickness during pregnancy, poor food cooking conditions, poor self-management, difficulty in altering life habits, and an inadequate understanding of GDM.

### Numbers of GDM risk factors

As stated in the inclusion criteria, we stratified the GDM risk factors screened in the first trimester and analyzed the results of abnormal glucose screening in the second trimester. Then we discerned a positive relationship between the risk for GDM and the number of risk factors (*p* < 0.05). In the logistic regression analysis, potential confounding variables such as age, gravidity and parity were included to adjust for their effects on the outcome.

**Figure 2:**
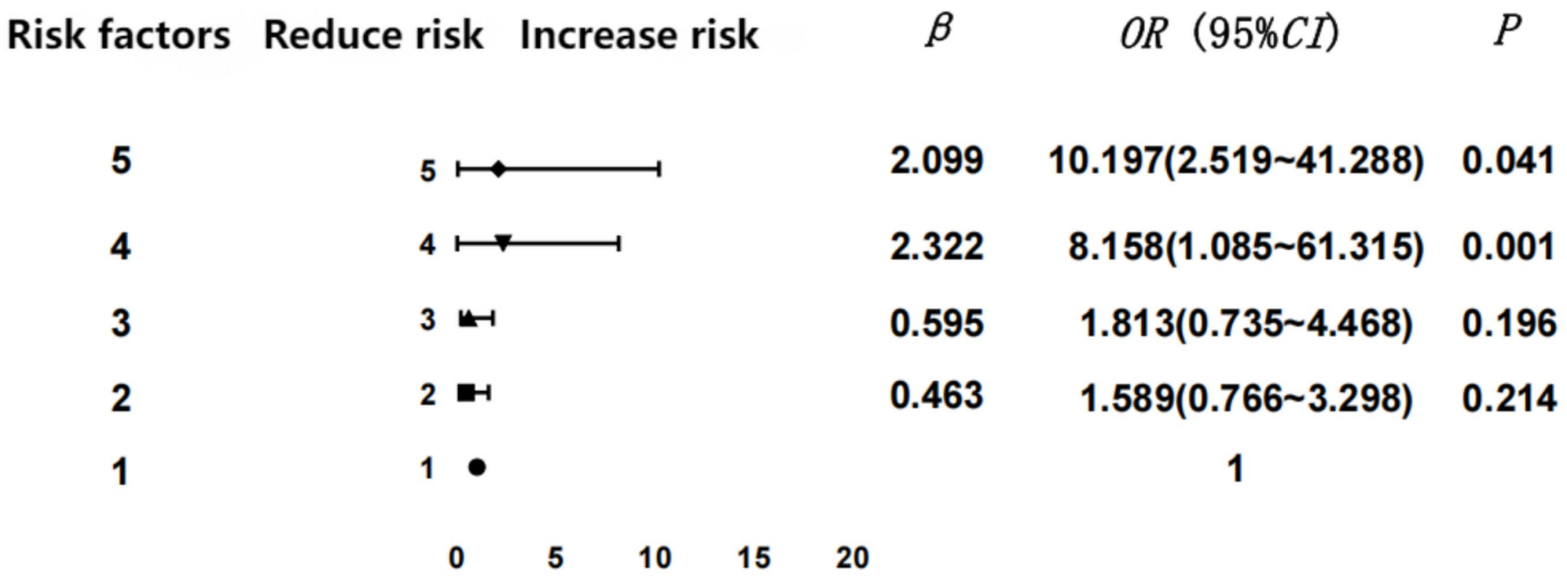
Forest plot reflecting the regression analysis of GDM risk factors.

To clarify the regression analysis results, we performed statistical analysis via GraphPad Prism 9.0 software to obtain a forest graph of the correlation between the number of GDM risk factors and GDM. Our results indicated that the incidence of GDM rose commensurate with the increase in the numbers of risk factors. Four or more risk factors were significantly associated with GDM (the forest plot reflecting the regression analysis of the GDM risk factors is shown in Figure 2).

## Discussion

### Principal findings

#### Pregnancy log management for the prevention of GDM

The incidence of GDM in the second trimester was significantly lower in the observational group than in the control group. Log recording strengthen the management of pregnant women’s dietary, physical activity, sleep patterns and other behavioral habits. Through pregnancy log records and management plans, reasonable dietary habits, appropriate exercise, and good self-management can be achieved, thus reducing sugar load and promoting glucose metabolism. It is very important to educate high-risk populations in advance. Log records provided real-time feedback and improved self-awareness, which could specifically reduce the incidence of GDM. On the basis of the Planned Behavior Theory, an individual’s motivation and plans are usually the foundation of behavioral change. According to the theory of “knowledge-belief-action,” it is beneficial to implement measures for pregnant women by correcting their lifestyle and improving their compliance in a timely fashion (13).

#### Influence of risk factors on the incidence of GDM

Pregnant women with four or more risk factors comprise a vulnerable population. Attention should therefore be given to those women with high fasting blood glucose and risk factors in early pregnancy, and further glucose tolerance screening and early interventional management should subsequently be conducted to prevent the occurrence of GDM (14). A family history of diabetes is also an independent risk factor for GDM in primiparas (15), and the risk of GDM in pregnant women over 35 years of age is greater than that under 30 years of age (16).

Excluding extreme cases, the BMI of the subjects in this study was relatively good, indicating that pregnant women had a heightened awareness of weight control before pregnancy and were satisfied with their BMI; however, the rate of weight gain at 28 weeks of gestation was relatively low. We recommend that self-management and education for pregnant women should be strengthened to control their BMI and reduce the incidence of GDM (17).

### Influence of daily behaviors on the onset of GDM

#### Dietary habits

As researchers have reported, individualized nursing guidance and dietary intervention can effectively prevent GDM and reduce adverse pregnancy outcomes in GDM patients (18). Our patients’ poor eating habits principally included excessive intake of high-oil, high-sugar, and high-fat foods, incorrect cooking methods, and excessive consumption of staple foods. During their early pregnancy education, pregnant women were instructed on how to exchange food for a reasonable diet to ensure that they can independently manage their own diets.

#### Physical activity

It was revealed that light physical activity in the first trimester of pregnancy was associated with a > 20% reduction in the risk of GDM, results similar to those of the present study (19). Mottola et al. also reported that GDM was effectively managed and prevented by regular exercise (20). Regular physical activity during pregnancy can prevent weight gain, GDM, preeclampsia, and cesarean delivery, and improve mental health (21). Appropriate physical activity can additionally reduce serum insulin levels, regulate insulin receptors, improve carbohydrate utilization and insulin resistance (IR), and then normalize blood glucose levels. Physical activity also reduces oxidative stress and systemic chronic inflammatory reactions; lowers the concentrations of inflammatory cytokines, C-reactive protein, and tumor necrosis factor; and delays the insulin demand of GDM patients (22).

#### Sleep patterns

Sleep duration and conditions of pregnant women constituted influencing factors. Sleep patterns were a key part of a pregnant woman’s lifestyle. Facco et al. reported that pregnant women whose sleep time was too brief (<7 h) in the second trimester were more likely to develop GDM (23). This was also reflected in the present study.

#### Smoking

Smoking can precipitate inflammation in the body and impair the internal environment. The influence of smoking on blood glucose may be attributed to the increase in glucotropic hormones and IR caused by harmful components such as nicotine in tobacco (24). The authors of a prospective study in Korea analyzed pregnant women with different numbers of smoking years, and reported that the incidence of GDM rose with increasing years of smoking, and that the risk of requiring insulin therapy was also augmented (25). We therefore recommend counseling families and restricting the use of tobacco in public places.

#### Pregnancy weight gain

Good pregnancy management is beneficial for controlling weight gain during pregnancy and is conducive to the maternal and infant health. Body weight and body mass index are important indicators in clinical practice. According to MacDonald (26), the incidence of GDM increases 23% for every standard deviation of weight gain in the first trimester in normal-weight women, exceeding the predicted weight gain. Some scholars also reported that weight gain in early pregnancy was associated with an increased risk of GDM (27). The proliferation of adipocytes in obese patients also leads to a relative decrease in insulin receptors per unit surface area and diminishes insulin sensitivity. Moreover, the secretion of leptin, interleukin 6, tumor necrosis factor-*α*, adiponectin, and other inflammatory factors are also induced; and these molecules aggravate IR (28).

#### Strengths and limitations

This study encompassed all controllable risk factors recognized in GDM, and exploited the standard instructional courses so as to establish and optimize educational content and process. At early pregnancy, we evaluated the influence of active participation by women in the effective intervention of high-risk factors on the incidence of GDM. We also observed the effects of screening and management of fixed high-risk factors during pregnancy on dynamic high-risk factors.

As a single-center study, our subjects were all from the same hospital, and the sample size was not large. Thus, this work necessitates multi-center and larger-sample studies in the future. There might be confounding bias in the study. Furthermore, lacking of blinding for outcome assessors risks detection bias is another limitation.

### Interpretation

#### Effects of maternal initiative and compliance management

It has been suggested that good compliance during pregnancy, effective questioning, and active changes in lifestyle habits can prevent the onset of GDM. Scholars posited that pregnant women with stronger self-management abilities reflect more planning and better compliance with their dietary management (29). The reasons for poor compliance in our observational group were the relatively weak awareness of active management, and the inability to recognize potential dangers or existing health hazards. Therefore, to reduce the risk of GDM, maternal initiative and compliance need to be enhanced.

#### Necessity of screening for GDM risk factors in early pregnancy

It is necessary to screen pregnant women with a high risk of GDM for diabetes in early pregnancy, and those women with abnormal blood glucose should be included in the diabetic population as soon as possible. This suggests that obstetricians should in the future focus on high-risk groups with respect to GDM.

Early management or treatment of pregnant women with abnormal screening results can effectively reduce the incidence of GDM and thereby improve adverse pregnancy outcomes. The early identification and appropriate management of GDM were demonstrated to constitute significant factors in reducing the burden of adverse perinatal outcomes in the UK (30). In Australia, pregnant women at high risk for GDM are recommended to undergo blood glucose screening in the first trimester before 24 weeks of gestation, and the authors of one prospective study even suggested that pregnant women screened in the first trimester show improved pregnancy outcomes relative to those only screened in the second trimester (31). Thus, the need for screening of GDM risk factors in early pregnancy certainly requires further clarification.

## Conclusion

Log records and management measures have an effective impact on reducing the incidence of GDM. Dietary habits, physical activity, sleep patterns, exposure to passive smoking, and weight gain during pregnancy are among the risk factors associated with GDM. Maternal lifestyle is a critical determinant influencing the incidence of GDM.

## Data Availability

The original contributions presented in the study are included in the article/supplementary material, further inquiries can be directed to the corresponding authors.
